# Hypocalcemia as an Independent Cause of Esophageal Dysphagia: A Case Report

**DOI:** 10.7759/cureus.70549

**Published:** 2024-09-30

**Authors:** Muhammad Zain Akhtar, Shakeel Ahmed, Zahid Idrees, Nadine Sakrana

**Affiliations:** 1 Department of Medicine, Royal Preston Hospital, Preston, GBR

**Keywords:** calcium supplementation, dysphagia, electrolyte imbalance, esophageal dysmotility, hypocalcemia, hypomagnesemia, loop diuretics, magnesium therapy, parathyroid hormone

## Abstract

Dysphagia, or difficulty swallowing, can result from esophageal motility disorders and is often linked to hypocalcemia. Calcium is essential for the proper function of pharyngeal and esophageal muscles, and low calcium levels can impair swallowing. Additionally, certain diuretics may worsen this by increasing calcium and magnesium loss, complicating the management of hypocalcemia. In this case, an 82-year-old male presented with a sudden onset of dysphagia, which had worsened over one week. He was intolerant to both solids and liquids and had a complex medical history, including non-erosive gastritis and previous benign adenoma surgery. Magnetic resonance imaging (MRI) of the brain ruled out acute stroke. On admission, he had severe hypocalcemia (serum calcium level of 1.19 mmol/L) and hypomagnesemia (serum magnesium level of 0.17 mmol/L). A mildly elevated parathyroid hormone (PTH) level of 7.5 pmol/L suggested a compensatory response. Despite intravenous (IV) calcium administration, his calcium levels showed only slight improvement. Hypomagnesemia contributed to resistance to calcium therapy, necessitating IV magnesium. His use of furosemide at a dose of 40 mg per day, a diuretic that increases renal calcium excretion, likely exacerbated both conditions. After two days of IV calcium and magnesium, his serum calcium normalized to 1.96 mmol/L, and magnesium improved to 0.84 mmol/L.

Post-treatment, he showed marked improvement in dysphagia, regaining the ability to swallow both solids and liquids. The endoscopic examination of the upper gastrointestinal (GI) tract shows normal findings. The gastroesophageal junction and esophagus have healthy mucosa with no visible abnormalities such as lesions, strictures, or inflammation. The stomach appears normal, with smooth, intact mucosa and well-formed gastric folds. Some food debris is present but does not indicate any pathology. The resolution of symptoms with electrolyte correction confirmed that hypocalcemia was the primary cause of his dysphagia, which was challenging to manage due to hypomagnesemia. This case emphasizes the importance of considering electrolyte imbalances, particularly hypocalcemia, as potential reversible causes of severe dysphagia. The patient’s improvement in swallowing function with calcium correction supports the hypothesis that these electrolyte disturbances significantly contributed to his symptoms. This case highlights the crucial role of calcium in esophageal motility and emphasizes the need to evaluate and correct electrolyte imbalances in acute dysphagia. This case also highlights the role of magnesium in overcoming resistance to calcium supplementation in cases of severe hypocalcemia. Further research could improve the understanding and management of similar cases.

## Introduction

Calcium is central to most cellular functions and acts as a fundamental secondary messenger of critical importance in cellular signaling processes. It is also an indispensable component that governs skeletal, smooth, and cardiac muscle contractility. In smooth muscles, calcium initiates contraction by binding to calmodulin, which, in turn, activates the enzyme myosin light chain kinase. This activated enzyme phosphorylates the myosin filaments, resulting in smooth muscle contraction. The homeostasis of calcium in the body is regulated by the parathyroid hormone (PTH) and vitamin D feedback loop [1]. Hypocalcemia is when the serum calcium levels plummet to less than the normal range of 2.1 to 2.6 mmol/L [2]. It can be acute or chronic, and its severity can range from asymptomatic to life-threatening crises. In this case, dysphagia was due to hypocalcemia, as decreased serum calcium levels alter the contractility of smooth muscles, affecting autonomic ganglia. This can cause abdominal pain and dyspnea [2]. It can also manifest in the cardiac system as QT interval prolongation and abnormal T waves in electrocardiogram (ECG). Such a hypocalcemic condition can be life-threatening, especially in older patients [3].

Hypocalcemia is often linked to hypomagnesemia, where serum magnesium levels fall below 0.66 mmol/L, with clinical symptoms typically appearing when levels drop below 0.50 mmol/L [4]. Magnesium is essential for key biochemical processes, including protein and nucleic acid synthesis. It plays a vital role in over 300 enzymatic reactions in the body, such as facilitating the binding of adenosine triphosphate (ATP) to enzymes like pyruvate kinase and ATPase [5]. Clinically relevant signs of hypomagnesemia include tremors, tetany, convulsions, psychiatric problems like delirium, and cardiac manifestations like arrhythmias. Hypomagnesemia can also make hypocalcemia challenging to manage, as the latter is often unresponsive to calcium therapy alone and requires magnesium supplementation [4,6].

Hypocalcemia and hypomagnesemia are intricately linked, mainly through their biochemical mechanisms. Magnesium is essential for the synthesizing and secretion of PTH, which is critical in maintaining calcium homeostasis [1]. When magnesium levels are low, PTH secretion is impaired, leading to decreased mobilization of calcium from bone stores and reduced renal reabsorption of calcium, ultimately resulting in hypocalcemia. Additionally, magnesium is vital for converting vitamin D into its active form, calcitriol [1,4]. Low magnesium levels can hinder this conversion, leading to decreased intestinal absorption of calcium. As a result, the body becomes less efficient at absorbing dietary calcium, further contributing to low serum calcium levels. Calcium and magnesium are crucial for muscle contraction and neurotransmitter release at the cellular level. A magnesium deficiency can increase neuromuscular excitability, leading to symptoms such as muscle cramps and tetany. This interplay highlights the importance of monitoring both electrolytes; treating hypomagnesemia can restore normal calcium levels and improve overall metabolic function [4,6].

The manifestation of dysphagia (difficulty in swallowing) in an older patient is caused by hypocalcemia. Dysphagia is observed to be more prevalent in the older population than in the general population due to the number of comorbid conditions in older people. In the USA, an estimated 15-22% of community-dwelling people over the age of 50 have dysphagia, while 40-60% of those in nursing homes suffer from swallowing problems [7]. Dysphagia can manifest itself as oropharyngeal or esophageal. In oropharyngeal dysphagia, hindrance occurs to food movement from the pharynx to the esophagus. In contrast, in esophageal dysphagia, patients have difficulty passing food from the esophagus to the stomach [7]. The objective of this case study is to highlight hypocalcemia as the reversible cause of esophageal dysphagia in older patients. By detailing the diagnosis and treatment of a patient with severe dysphagia due to these electrolyte imbalances, the study emphasizes the importance of recognizing and correcting these disturbances. It also underscores the role of magnesium in overcoming resistance to calcium therapy in managing severe hypocalcemia.

## Case presentation

An 82-year-old patient presented with severe esophageal dysphagia, which significantly impacted their ability to swallow. He was intolerant to both solids and liquids for a week. The patient had previous reports of mucosal thickening of the gastroesophageal junction and non-erosive gastritis with signs of chronic inflammation. He also had undergone prior surgery for benign adenoma with low-grade dysplasia and coronary artery bypass graft (CABG).

**Figure 1:**
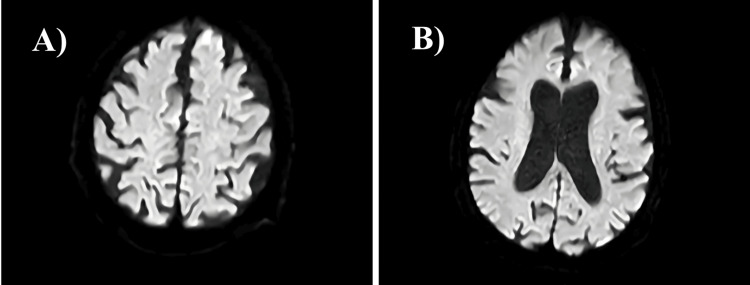
Images A and B show axial magnetic resonance imaging (MRI) scans from an examination of an 82-year-old male with no acute abnormalities, demonstrating age-related involutional changes.

Magnetic resonance imaging (MRI) of his brain was performed to rule out acute stroke. The axial MRI scans revealed no acute abnormalities but demonstrated typical age-related involutional changes (Figures 1A, 1B).

Baseline investigation showed hypocalcemia, where it was revealed that the patient had critically low levels of calcium (1.19 mmol/L) (Table 1). Additionally, the PTH levels were mildly high (7.5 pmol/L). The patient was administered intravenous (IV) calcium; however, the levels remained unresponsive to the IV therapy. Since the hypocalcemia was resistant to treatment, his magnesium levels were tested and found to be abnormally low (0.17 mmol/L), confirming co-existing hypomagnesemia. It was believed that the esophageal dysphagia was due to severe hypocalcemia, which was difficult to manage due to the co-existing hypomagnesemia. Additionally, the patient was on long-term furosemide at a dose of 40 mg per day, which is known to cause electrolyte imbalances. Hence, the patient was administered IV magnesium and calcium for two days, increasing calcium (1.96 mmol/L) and magnesium (0.84 mmol/L) levels (Table 1). Subsequently, the patient was put on oral calcium and magnesium supplements to elevate the respective electrolyte levels further. The patient continued being responsive to the therapy and exhibited symptomatic improvement. The patient was kept under observation for a few more days, but he remained stable and continued to eat and drink effortlessly.

**Table 1:** Clinical parameters and reference values of an 82-year-old patient with electrolyte imbalances.

Parameter	Pre-treatment values	Post-treatment values	Normal values
Age (82 years)			
Duration tolerance (one week)			
Calcium level	1.19 mmol/L	1.96 mmol/L	2.1-2.6 mmol/L
Magnesium level	0.17 mmol/L	0.84 mmol/L	0.7-1.0 mmol/L
Parathyroid hormone level	7.5 pmol/L	-	1.6-6.9 pmol/L

**Figure 2:**
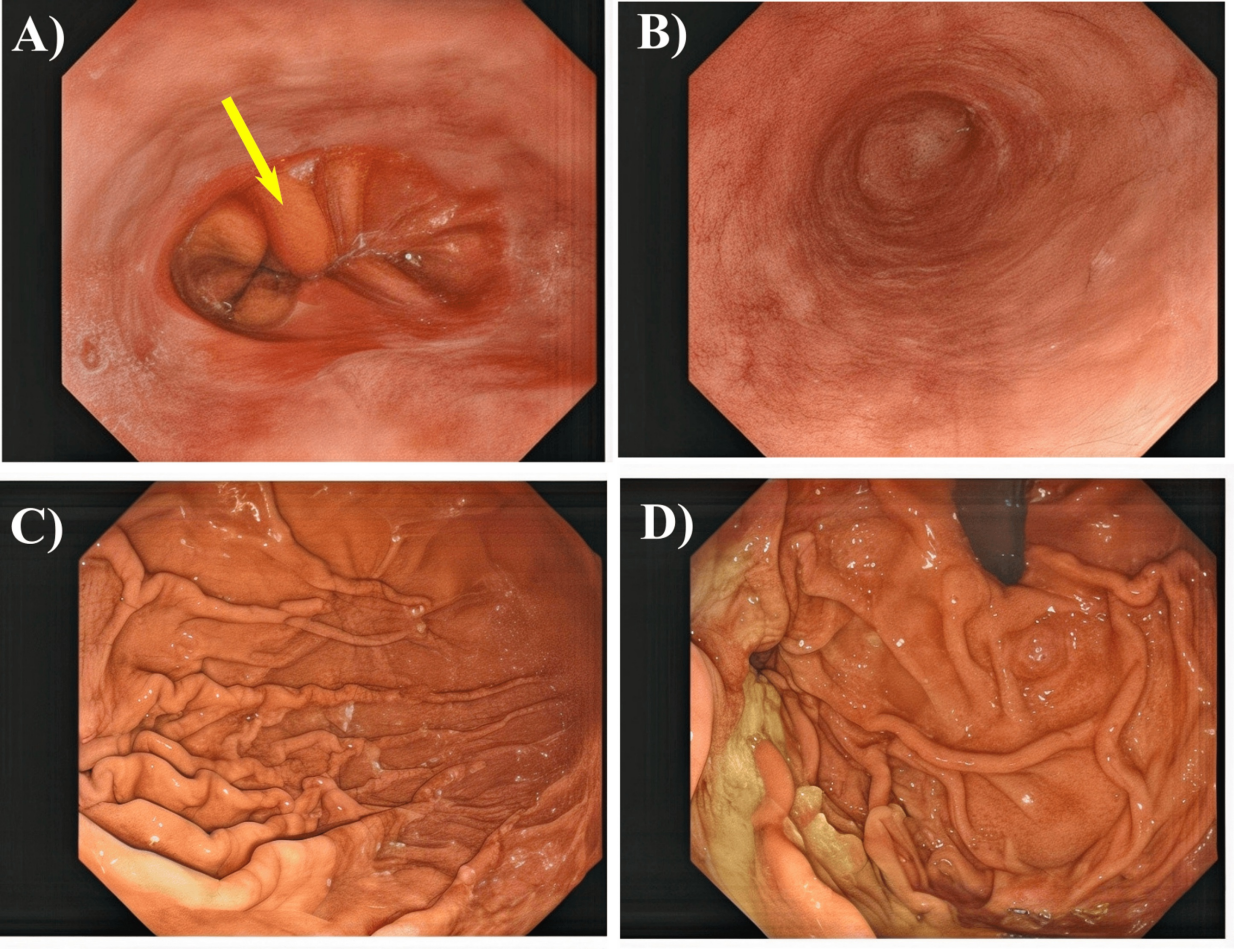
The endoscopy images show a normal gastroesophageal junction with healthy mucosa and a clear boundary between the esophagus and stomach (A). The esophagus appears normal, with a smooth mucosal surface and no visible abnormalities (B). The stomach is normal with a smooth, intact mucosal surface, though some visible food debris is present, not indicative of any pathology (C and D).

The endoscopic views from an upper gastrointestinal (GI) tract examination show various sections of the GI tract (Figure 2). The gastroesophageal junction appears normal with no visible abnormalities (Figure 2A). The arrow points to the intersection between the esophagus and the stomach, highlighting where the two structures meet. The mucosa is healthy, with a clear boundary between the esophageal and gastric tissues, showing no signs of lesions, strictures, or inflammation (Figure 2A). The esophagus also appears normal, with a smooth mucosal surface, and shows no visible abnormalities such as erosions and ulcers (Figure 2B). The mucosa is intact with no sign of inflammation or any other pathological changes (Figure 2B). Different views of the stomach show a normal mucosal surface that is smooth and intact (Figures 2C, 2D). Some visible food debris is noted, but this is common during endoscopy and does not indicate any pathology (Figures 2C, 2D). Overall, the findings indicate a normal upper GI tract examination. This case highlights the improvement of esophageal dysphagia due to severe hypocalcemia, which was initially resistant to IV calcium supplementation due to underlying hypomagnesemia but became responsive after magnesium therapy.

## Discussion

Dysphagia, or difficulty swallowing, is a complex symptom arising from various underlying conditions, including electrolyte imbalances like hypocalcemia. In this case, dysphagia was directly linked to severe hypocalcemia, which underscores the critical role of normal serum calcium levels in maintaining esophageal and pharyngeal muscle function. The present case highlights the often-overlooked connection between these electrolyte disturbances and esophageal motility disorders, demonstrating the need for a comprehensive evaluation of serum electrolytes in patients with dysphagia.

Normal serum calcium is essential for pharyngeal and esophageal muscle function. Smooth muscles, with limited internal calcium stores, depend on extracellular calcium to initiate and maintain contraction via calcium influx and the RhoA kinase pathway. Low serum calcium in this patient impaired esophageal muscle function, leading to dysphagia [8]. The drug-induced hypocalcemia can be easily missed in clinical settings due to the multifactorial nature of its origin. Loop diuretics like furosemide increase renal calcium excretion and cause volume contraction and alkalosis, contributing to hypocalcemia, hypomagnesemia, and esophageal dysmotility [9].

The present case study is a prime example of hypocalcemia that was difficult to manage due to severe hypomagnesemia. Hypocalcemia and hypomagnesemia can have similar symptoms due to the influence of magnesium in the transport of calcium over the cell membrane. Hypomagnesemia can also lead to hypokalemia in some cases since magnesium is essential for reabsorbing potassium in the renal tubules. Hence, the metabolic effects of magnesium may cause hypocalcemia and hypokalemia to become resistant to the treatment in the face of hypomagnesemia. In this case, hypocalcemia became resistant to therapy because of co-existing hypomagnesemia, which required correction of magnesium through IV or oral treatment to restore calcium levels [10].

Furthermore, hypomagnesemia can impair PTH function, further complicating hypocalcemia management. PTH facilitates magnesium reabsorption in the kidneys, and a mild decrease in serum magnesium levels induces further secretion of PTH by the parathyroid glands. However, if the serum magnesium levels fall below 1.2 mg/dL or 0.06 mmol/L, it leads to a decline in the PTH secretion and ultimately causes hypocalcemia [11]. There are several factors and many pathways that can precipitate hypocalcemia. It can be post-operative conditions, drug-induced, or due to an electrolyte imbalance of major ions like magnesium. An example is the case study of a 58-year-old male who was experiencing stridor, dysphagia, and dysphonia simultaneously as a result of previous thyroid surgery. The screening before a planned endoscopy unveiled severe hypocalcemia, and he was immediately put on IV calcium for 48 hours. He was given high-dose oral calcium supplements; his symptoms subsided later [3]. Our case is similar in that hypocalcemia was the primary cause of dysphagia, but in this case, the treatment required magnesium correction before the hypocalcemia could be resolved.

Another scenario that precipitates hypocalcemia is the unrecognized interactions among prescribed drugs. A 65-year-old male patient, who was on rivaroxaban, esomeprazole, and fluconazole with a history of multiple myeloma, presented with severe neurological and GI symptoms. His blood analysis revealed hypocalcemia, hypokalemia, and hypomagnesemia. The metabolic alkalosis and electrolyte imbalances were attributed to drug interactions between fluconazole and esomeprazole, which led to severe hypomagnesemia, causing secondary hypocalcemia and hypokalemia [10]. In the present case study, while dysphagia was due to severe hypocalcemia, the management of hypocalcemia was complicated by underlying hypomagnesemia. Hypomagnesemia did not directly cause the dysphagia but made the hypocalcemia resistant to calcium therapy. Once magnesium levels were corrected, calcium supplementation became effective, and the patient's dysphagia improved. This highlights the importance of addressing hypomagnesemia to manage hypocalcemia in patients with esophageal dysphagia.

## Conclusions

This case study exemplifies that severe dysphagia was caused by hypocalcemia, highlighting the critical role of calcium in maintaining proper esophageal function. The patient’s dysphagia, which was initially resistant to calcium treatment, was challenging to manage because of underlying hypomagnesemia, which impaired the effectiveness of calcium therapy. IV magnesium was necessary to resolve the hypocalcemia, allowing for successful treatment of the dysphagia. This demonstrates that effective management of such cases requires addressing co-existing hypomagnesemia to treat hypocalcemia-induced dysphagia. Additionally, the case emphasizes the impact of drug-induced factors, such as the effects of loop diuretics, on both calcium and magnesium levels. Despite the patient's complex medical history, including GI disorders, targeted calcium and magnesium replacement therapy significantly improved swallowing function. This underscores the value of a thorough assessment and individualized treatment strategy in managing dysphagia related to hypocalcemia, improving patient outcomes through careful electrolyte management.
